# Discordant Timing of Hypoglycemic Agent Screening Causing Delayed Diagnosis of Sulfonylurea-Induced Hypoglycemia

**DOI:** 10.1016/j.aace.2021.12.006

**Published:** 2022-01-03

**Authors:** Andrew Folick, Cheng Cheng, Simon N. Chu, Jonathan W. Rick, Robert J. Rushakoff

**Affiliations:** Division of Endocrinology and Metabolism, Department of Medicine, University of California, San Francisco, California

**Keywords:** Sulfonylurea, Hypoglycemia, factitious disorder, drug-induced hypoglycemia, glipizide

## Abstract

**Background:**

Oral hypoglycemic agents are a frequent cause of hypoglycemia in nondiabetic people. Here, we report a case of recurrent hypoglycemia caused by glipizide, in which diagnosis was delayed because of a combination of delayed hypoglycemic agent screening and low sensitivity of the hypoglycemic agent screening panel used.

**Case Report:**

A 66-year-old woman repeatedly presented with symptomatic hypoglycemia. At the first presentation, the serum glucose level was 40 mg/dL (2.2 mmol/L), C-peptide level was 13.1 ng/mL (0.8-3.1 ng/mL), proinsulin level was 96.9 pmol/L (<18.8 pmol/L), and insulin level was 164 mU/L (<17 mU/L). An initial hypoglycemic agent screening, performed 24 hours after admission, yielded a negative result, leading to prolonged and recurrent hospitalizations for workup and localization of insulinoma. A hypoglycemic agent screening at a subsequent presentation, concordant with hypoglycemia, yielded a positive result for glipizide, which was at a level of 320 ng/mL (reporting limit, 40 ng/mL). An examination of the patient’s home medications revealed a container, labeled as benztropine, containing glipizide tablets. After the diagnosis of glipizide-induced hypoglycemia, the patient had no further episodes of hypoglycemia.

**Discussion:**

The failure to detect glipizide using the initial hypoglycemia agent assay was likely because of a combination of a delay in the initial screening and low sensitivity of the assay for glipizide compared with that of other available assays. Here, we discuss important considerations for the interpretation of hypoglycemic agent screening in the diagnosis of hypoglycemia, including the timing of collection and reporting, pharmacokinetics of culprit agents, and sensitivity of the hypoglycemic agent panel used.

**Conclusion:**

Screening tests for hypoglycemic agents are necessary for the evaluation of hypoglycemia because their biochemical evaluation may be indistinguishable from that of insulinoma.

## Introduction

Oral hypoglycemic agents are a frequent cause of hypoglycemia in nondiabetic people. We report a case of recurrent hypoglycemia in a patient without diabetes who was subsequently found to be taking glipizide. The diagnosis was delayed due to a combination of delayed hypoglycemic agent screening and low sensitivity of the hypoglycemic agent screening panel used. We discuss how to avoid these problems in diagnosis.

## Case Report

A 66-year-old woman, with a past medical history significant for bipolar disorder, anxiety, depression, and essential tremor, underwent multiple admissions for recurrent hypoglycemia.

At the first presentation, the patient was found unconscious by emergency medical services, with a glucose level of 40 mg/dL, and was revived using dextrose on field. At presentation in the emergency department, the serum glucose level was 40 mg/dL (2.2 mmol/L), C-peptide level was 13.1 ng/mL (0.8-3.1 ng/mL), proinsulin level was 96.9 pmol/L (<18.8 pmol/L), insulin level was 164 mU/L (<17 mU/L), β hydroxybutyrate level was 0.1 mmol/L (<0.28 mmol/L), and insulin autoantibody level was <0.4 U/mL (<0.4 U/mL). On hospital days 2 and 3, hypoglycemic agent screening panels (NMS Labs) showed that the patient was negative for the presence of sulfonylurea or meglitinide agents (Fig. *A*). Notably, the hypoglycemic agent screening’s results were not available for 7 days. A 24-hour supervised fast was performed with lowest fingerstick glucose 75 mg/dL (4.1 mmol/L). Magnetic resonance imaging of the abdomen showed an unremarkable pancreas and biliary tree. The patient was discharged but readmitted 1 day later, after waking up with shaking and anxiety, with a fingerstick glucose level 42 mg/dL. At presentation, the glucose level was 31 mg/dL (1.7 mmol/L), C-peptide level was 18.2 ng/mL (0.8-3.1 ng/mL), proinsulin level was 189.1 pmol/L (<18.8 pmol/L), insulin level was 296 mU/L (<17 mU/L), and β hydroxybutyrate level was 0.2 mmol/L (<0.28 mmol/L); a hypoglycemic agent screening (NMS Labs, results returned in 9 days) yielded a positive result for glipizide, which was at a level of 320 ng/mL (Fig. *A*). However, based on the previous negative hypoglycemic agent screening performed during the first admission, the patient was transferred for further workup for recurrent hypoglycemia and the consideration of endoscopic ultrasound to evaluate for insulinoma. Upon transfer, the patient’s home medications were manually inspected, and a pill bottle, labeled as “benztropine 0.5 mg 2 time daily,” containing generic glipizide tablets was found. A 72-hour fast was performed, ending at 72 hours with a glucose level of 60 mg/dL (3.3 mmol/L), C-peptide level of 0.5 ng/mL (0.8-3.5 ng/mL), proinsulin level of 6.9 pmol/L (3.6-22 pmol/L), insulin level of 3.4 mU/L (3.0-19.0 mU/L), β hydroxybutyrate level of 4.41 mmol/L (0.02-0.27 mmol/L), and negative serum hypoglycemic agent screening result (ARUP Laboratories, results returned in 4 days). The patient was discharged with a diagnosis of glipizide-induced hypoglycemia.

**Fig:**
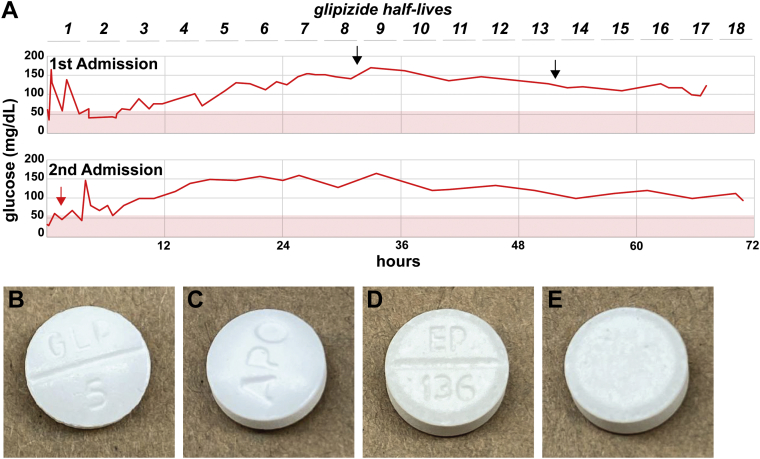
The timing of hypoglycemic agent screening revealed glipizide tablets as the cause of hypoglycemia. *A*, The laboratory testing result for hypoglycemic agents was negative (black arrows) during the first admission when testing was delayed for approximately 24 hours, during which time multiple half-lives of sulfonylurea degradation would have been predicted. The testing result was positive for glipizide (red arrow), which was at 320 ng/mL, during the subsequent admission when performed at presentation. *B* through *E*, Similar appearance of 5-mg glipizide tablets (Apotex) (*B, C*) and 0.5-mg benztropine tablets (Leading Pharma) (*D*, *E*).

## Discussion

Screening tests for hypoglycemic agents, commonly including sulfonylureas and meglitinides, are necessary for the evaluation of hypoglycemia but may be subject to errors in interpretation. These tests are not comprehensive, potentially lacking new agents and agents that have not been approved by the U.S. Food and Drug Administration.^1^^,^^2^ Furthermore, some nondiabetes medications are known to potentially induced hypoglycemia, and these are not included in available screening assays.^3^ One study found that 37% of nondiabetic patients with a low glucose level and inappropriately high insulin and C-peptide levels were positive for a sulfonylurea agent.^4^ Assays using liquid chromatography tandem mass spectrometry, such as that currently used by the Mayo Clinic clinical laboratory and ARUP Laboratories, offer the most sensitive detection of sulfonylurea and meglitinide agents.^5^ However, many hypoglycemic agents are rapidly metabolized, and plasma levels that cause hypoglycemia can decrease to levels below the threshold of detection in hours. Specific to this case, the half-life of glipizide has been estimated to be 2 to 7 hours, varying by individual, with a mean half-life of approximately 4 hours.^6^ Standard immediate-release glipizide at therapeutic doses results in plasma concentrations peaking approximately 2 hours after ingestion, with levels in the range of 200 to 1000 ng/mL.^7^ The detection limit for glipizide is 3 ng/mL in the Mayo Clinic assay, 4 ng/mL in the ARUP assay, and 40 ng/mL on the NMS Labs assay, which was used during the first 2 admissions. Thus, the doses of glipizide sufficient for inducing hypoglycemia may be undetectable within hours, and this varies depending on the detection limit of the assay. For example, an initial glipizide level of 320 ng/mL (the level obtained for this patient, concurrent with hypoglycemia), assuming a 4-hour half-life, would be undetectable after 12 hours with the NMS assay and 27 hours with the Mayo Clinic assay. Urine assays detecting sulfonylurea or meglitinide metabolites offer a longer window of detection but are not routinely available.^8^ Furthermore, the time frame of reporting by reference laboratories may not allow for the results to in aid timely clinical decision making.^8^

### Results, Interpretation, and Patient Application

At presentation during both the admissions for hypoglycemia, the Whipple triad (low plasma glucose concentration, signs and symptoms of hypoglycemia, and resolution of symptoms with treatment of hypoglycemia) was documented, indicating the necessity of workup for hypoglycemia. The elevations in the C-peptide, insulin, and proinsulin levels, concurrent with hypoglycemia, indicate that hypoglycemia was due to hyperinsulinemia. The elevation in the C-peptide level ruled out exogenous insulin as the culprit. Although the molar ratio of insulin to C-peptide is useful in the diagnosis of factitious hypoglycemia caused by exogenous insulin, hypoglycemia caused by insulin secretagogues may be biochemically indistinguishable from insulinoma unless the causative agent is identified.^9^ The detection of glipizide at a level sufficient to induce hypoglycemia, concurrent with documented symptomatic hypoglycemia, is consistent with glipizide-induced hypoglycemia. The previous sulfonylurea agent screening results were likely negative because of discordant timing and the relatively high reporting limit of the assay used (Fig. *A*). Sulfonylurea and meglitinide medications can cause hypoglycemia in nondiabetic people in cases of both surreptitious use and a pharmacy error. The prevalence of pharmacy errors is predicted to vary between <1% to >6%.^10^ Notably, glipizide and benztropine are similar-appearing circular white tablets, which may contribute to a pharmacy error (Fig. *B*).

### Patient Outcome

In this case, the patient underwent prolonged hospitalization and workup for recurrent hypoglycemia, which might have been avoided with earlier, appropriately timed hypoglycemic agent screening. After being informed that hypoglycemia was caused by glipizide, the patient appeared inappropriately unconcerned. A psychiatric evaluation was performed, but because of the possibility of a pharmacy error, there was no definitive diagnosis of factitious disorder. The patient has not had subsequent episodes of hypoglycemia.

## Conclusion

We emphasized that hypoglycemic agent screening should be routinely performed at the initial presentation, ideally at the emergency department, for hypoglycemia of an unknown cause. High-volume centers may consider the implementation of rapid on-site serum hypoglycemic agent screening to avoid unnecessarily prolonged hospitalization and workup.^8^ Consideration must be given to the timing of hypoglycemic agent screening in relation to presentation for documented hypoglycemia, the pharmacokinetics of potential culprit agents, and the sensitivity of the assay.
